# Supplemental Nutrition Assistance Program Policies and Food Insecurity

**DOI:** 10.1001/jamahealthforum.2025.5597

**Published:** 2025-12-12

**Authors:** Sriya Potluri, Atheendar S. Venkataramani, Scott A. Lorch, Nicholas Illenberger, Sameed Ahmed M. Khatana

**Affiliations:** 1 Perelman School of Medicine, University of Pennsylvania, Philadelphia; 2 The Leonard Davis Institute of Health Economics, University of Pennsylvania, Philadelphia; 3 Department of Medical Ethics and Health Policy, Perelman School of Medicine, University of Pennsylvania, Philadelphia; 4 Division of Neonatology, The Children’s Hospital of Philadelphia, Philadelphia, Pennsylvania; 5 Division of Biostatistics, Department of Population Health, New York University Grossman School of Medicine, New York; 6 Division of Cardiovascular Medicine, Perelman School of Medicine, University of Pennsylvania, Philadelphia

## Abstract

**Importance:**

Food insecurity (FI) is associated with poor health and has risen in the US. The Supplemental Nutrition Assistance Program (SNAP) is the largest US food-purchasing assistance program. Policies related to eligibility assessment and administrative burden that impact SNAP participation vary between states. How such policies influence FI is not well known.

**Objectives:**

To evaluate the association between changes in state SNAP policies and county FI rates.

**Design, Setting, and Participants:**

This repeated cross-sectional study used annual county-level FI estimates from the Feeding America Map the Meal Gap dataset, state-level SNAP policy data from the US Department of Agriculture from 2009 to 2019, and data on economic and demographic measures from the US Census Bureau for county residents. Data were analyzed from August 2024 to August 2025.

**Exposures:**

Changes in state SNAP policies from 2009 to 2019. Due to incomplete policy data, the analysis was not extended beyond 2019.

**Main Outcomes and Measures:**

County-level FI rates for individuals. An annual index of SNAP policy adoption was calculated, scaled from 0.1 to 10, with a higher level indicating a greater adoption of policies associated with SNAP participation. G-computation, a robust causal inference methodology, was used to evaluate the association between change in the SNAP index and state-level SNAP participation rates and county-level FI rates. The model accounted for demographic and clinical factors, state and year fixed effects, and baseline SNAP index levels.

**Results:**

Of a total of 3143 US counties, 3134 were included in the analysis. A 1-point increase in the SNAP policy index was associated with a 0.7–percentage point (pp; 95% CI, 0.3-1.2 pp; *P* = .002) higher state-level SNAP participation rate and a 0.1-pp (95% CI, 0.02-0.2 pp; *P* = .02) lower county-level FI rate from 2009 to 2019. In 2019, an estimated 6.5 million (95% CI, 3.8-9.1 million) fewer individuals would have experienced FI if all states had adopted policies equivalent to the most generous state in each year compared to if all states had adopted policies equivalent to the least generous state.

**Conclusions and Relevance:**

In this cross-sectional study, adoption of state-level policies associated with higher SNAP participation was also associated with lower county-level FI rates. Policies that lower barriers to SNAP participation may help address rising FI rates observed in 2022 and 2023.

## Key Points

**Question:**

Are state-level Supplemental Nutrition Assistance Program (SNAP) policies related to eligibility assessment and administrative burden, which differ between states, associated with county food insecurity rates in the US?

**Findings:**

In this cross-sectional study across 3134 US counties, state-level SNAP policies associated with greater SNAP participation were also associated with lower county-level food insecurity rates between 2009 and 2019.

**Meaning:**

State-level SNAP policies that minimize barriers to participation may play a role in improving food insecurity rates in the US.

## Teaser

This cross-sectional study evaluates the association between changes in state Supplemental Nutrition Assistance Program policies and county food insecurity rates.

## Introduction

Food insecurity (FI), the economic and social condition of inadequate consistent access to healthy food, is associated with worse health outcomes, including death.^1,2,3,4^ FI has risen recently in the US, and as of 2023, 13.5% of households experienced FI.^5^ Identifying strategies to address FI is essential for improving the health of vulnerable populations.

The Supplemental Nutrition Assistance Program (SNAP) is the largest government-funded program in the US providing food-purchasing assistance, with approximately 42.1 million participants in 2023.^6^ SNAP eligibility criteria and benefit levels are set nationally, but the program is state administered. While federal criteria limit SNAP participation to households with income 130% of the federal poverty limit (FPL) or lower, some states allow participation for households with income up to 200% of the FPL.^7^ Policies related to eligibility assessment and administrative burden can vary between states (eg, excluding vehicles from asset tests or fingerprinting participants). Such policies are associated with SNAP participation, as they can raise the income threshold to meet eligibility or increase the stigma of participation.^8,9,10,11^ Based on estimates from the University of Kentucky Center for Poverty Research, in 2023, among individuals with household income up to 200% of the FPL, state SNAP participation rates ranged from 18.7% (Wyoming) to 97.9% (Washington, DC).^12^ While SNAP participation is associated with lower FI on an individual or household level, whether such variation in state policies is associated with FI has not been well studied.^13^ Understanding this association is important for determining whether greater adoption of such policies may help address FI.

States with different SNAP policies may also differ in other economic and demographic factors associated with FI. For example, among residents of 9 states that have not enacted broad-based categorical eligibility (BBCE), a policy that can expand SNAP eligibility, as of 2025, or have done so in only a limited way, the proportion experiencing poverty is higher than the national average (13% vs 12.5%).^7,14^ Therefore, understanding the association between SNAP policies and FI requires an approach that addresses such potential confounding. This problem is particularly acute when SNAP policies and confounding characteristics themselves change over time. Therefore, using g-computation, a robust causal inference methodology that compared to traditional regression methods better accounts for time varying confounding, we studied whether adoption of state-level SNAP-related policies was associated with FI rates across the US between 2009 and 2019.

## Methods

### SNAP Policy Index

Data on state SNAP policies were obtained from the US Department of Agriculture’s SNAP policy database.^15^ Based on previous studies demonstrating an association with SNAP participation, we obtained monthly data on adoption of the following policies from 2009 to 2019: (1) BBCE, which allows for SNAP eligibility among households that qualify for noncash assistance through Temporary Assistance for Needy Families or state maintenance of effort funded benefits; (2) exemption of vehicles from asset tests; (3) average recertification period; (4) eligibility for legal noncitizens; (5) combined application processing with supplemental security income; (6) fingerprinting requirements; (7) online application availability; (8) proportion of benefits accounted for by electronic benefit transfer; and (9) simplified reporting of earnings.^8,9^ Due to incomplete policy data, the analysis was not extended beyond 2019. Continuous variables (eg, recertification period) were scaled from 0 to 1. For binary variables adopted for a portion of the year, the fraction of months in which the policy was implemented was used. As in previous analyses, an unweighted mean of these policies was then calculated, with a higher level indicating a greater adoption of policies associated with higher SNAP participation in each year from 2009 to 2019.^8,9^ This index was scaled to be between 0.1 and 10. Additional details regarding each policy and the index are in the eMethods in Supplement 1.

This analysis was considered exempt from review based on University of Pennsylvania institutional review board guidelines. All data used are publicly available. This study follows the Strengthening the Reporting of Observational Studies in Epidemiology (STROBE) reporting requirements.^16^

### FI and Other Data

County-level rates for individuals in food-insecure households were obtained from the Feeding America Map the Meal Gap dataset for 2009 to 2019.^17^ These estimates are produced first by modeling the association between state-level FI rates, obtained from the Current Population Survey (CPS), and different socioeconomic and demographic factors, from the American Community Survey (ACS), then using model coefficients and county-level ACS data to estimate county FI rates.^18^ A total of 9 sparsely populated counties did not have FI data available and were excluded. State-level FI rates were based on CPS estimates.

State SNAP participation was based on the number of individuals in households receiving SNAP obtained from University of Kentucky Center for Poverty Research national welfare data and the number with household income up to 200% of the FPL based on ACS data.^12^ We used 200% of the FPL as the denominator, as some states allow for participation up to this limit, and this allows for consideration of all potentially eligible individuals. SNAP participation from February 2019 was excluded due to a federal government shutdown. Other county and state-level economic and demographic measures were obtained from the US Census Bureau. Additionally, household-level FI rates, SNAP participation, and other economic, demographic, and household information were obtained from IPUMS-CPS.^19^

### Outcomes

The primary outcome was change in annual county-level FI associated with absolute change in the SNAP index from the baseline year (2009). A secondary outcome was state SNAP participation rates among individuals with low income.

### Statistical Analysis

We calculated summary measures of different demographic and economic variables across counties (at baseline), based on tertiles of the SNAP policy index in 2009. We also calculated annual summary measures of FI, SNAP participation, and the SNAP policy index. To examine the association between change in the SNAP index (from 2009) and change in FI rates, we used g-computation. In contrast to traditional regression, g-computation can isolate treatment effects in the presence of time-dependent confounding.^20^ As both FI and state SNAP policies are likely linked to economic and demographic factors, the analysis attempted to account for differences in these potential confounders between counties. We used county-level measures used within the Centers for Disease Control and Prevention’s Social Vulnerability Index that are associated with population-level health outcomes, including FI (eMethods in Supplement 1).^21,22,23^ The model also included health insurance coverage (among 18- to 64-year-old residents), county metropolitan status (based on the 2013 Rural-Urban Continuum Codes), as well as state and year fixed effects. In the g-computation process, baseline and nonbaseline (all years after 2009) levels of the outcome and each variable were modeled. Nonbaseline year models included 1-year lagged values of FI and other included covariates. The longitudinal relationship between SNAP policies, county-level FI, and other confounding variables was modeled using random forest algorithms. Observations in these models were weighted by county population. A marginal structural model was then estimated using generated outcomes drawn from the g-computation models. This model included FI as the dependent variable and baseline SNAP index, absolute change in SNAP index (for each year from 2009), and year fixed effects as the independent variables. State random intercepts were included to account for nesting of counties within states. Confidence intervals were constructed based on quantiles of the nonparametric cluster bootstrap (using 1000 iterations). This process was repeated, with state-level measures, for state-level models. Additional details on the g-computation process are in the eMethods in Supplement 1.

To examine the differential effects of each component of the SNAP policy index, we repeated the county- and state-level g-computation models using each of the individual policies that were used to construct the index in place of the overall index. We performed additional analyses under the longitudinal-modified treatment policy framework.^24^ This allowed us to assess the potential effect of targeted interventions on SNAP policies on county-level FI. Specifically, we estimated the difference in county-level FI rates across the US from 2009 to 2019 under 2 hypothetical scenarios: all states adopting the same policies as the most generous state for each year compared to all states adopting the policies of the least generous state. Index values used for each trajectory are listed in eTable 1 in Supplement 1. We also compared these trajectories with the observed population-weighted FI rate.

As the county FI estimates are model based, to assess the robustness of the estimates, we also fit g-computation models with state-level FI rates as the outcome. Additionally, we fit a logistic regression model with household-level FI as the outcome (among low-income households) using data from the 2009 to 2019 annual CPS food security supplement. This model included state and year fixed effects, individual-level (for head of household) and household-level covariates, and all state-level economic and demographic variables included in the g-computation models, as well as baseline SNAP index values and change in the SNAP index. Continuous variables were included as linear splines with 3 equally sized groups. A second logistic regression model with change in each individual policy was also fit. The models included person-level survey weights. State-level clustered robust standard errors were used. Additional details on the logistic regression models are in the eMethods in Supplement 1.

Summary measures and model estimates are presented as medians and IQRs or means and 95% CIs. All statistical tests were 2-sided, and a *P* < .05 was considered to be statistically significant. All analyses were performed using RStudio, version 4.2.2 (Posit), and data were analyzed from August 2024 to August 2025.

## Results

### County-Level Characteristics

**Figure 1.**
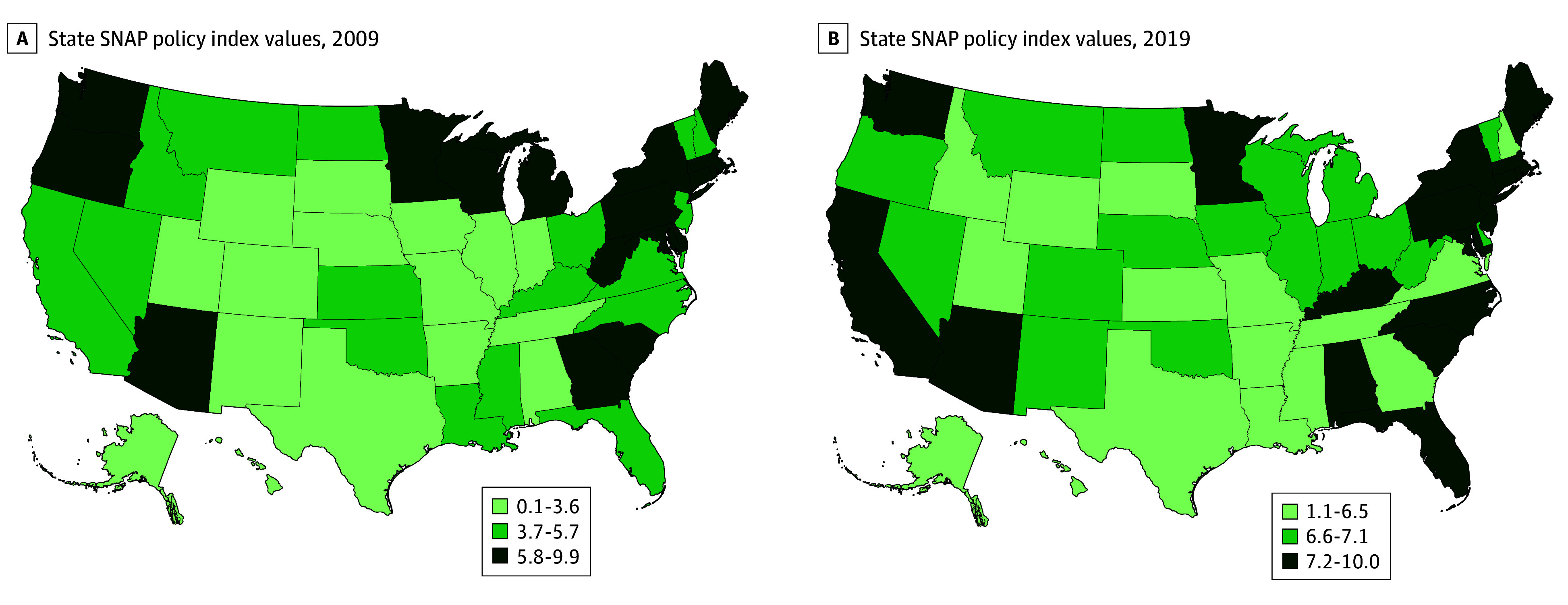
State Supplemental Nutrition Assistance Program (SNAP) Policy Index Values The SNAP policy index is scaled from 0.1 to 10, with a higher value indicating greater adoption of policies associated with SNAP participation. SNAP policy index and adoption of individual policies were obtained from the US Department of Agriculture’s SNAP policy database.^15^

Of a total of 3143 US counties, 3134 were included in the analysis. In 2009, the median (IQR) SNAP policy index value across all states was 4.7 (3.1-6.6). A map of US states based on tertile of the SNAP policy index at baseline is displayed in Figure 1 (tertile 1: 0.1-3.6; tertile 2: 3.7-5.7; tertile 3: 5.7-9.9). Summary measures of each SNAP policy for each tertile are listed in eTable 2 in Supplement 1. There was a higher median (IQR) proportion of residents living in poverty among counties in the first tertile at 15.9% (11.2%-17.7%) compared to counties in the second tertile at 4.4% (11.1%-16.8%) and third tertile at 13.0% (9.6%-16.8%) (Table 1). A smaller proportion of counties in the first tertile were metropolitan (375 of 1234 [30.4%]) compared to the second tertile (423 of 1109 [38.1%]) and third tertile (367 of 791 [46.5%]). Summary measures of other covariates for each tertile are listed in Table 1.

**Table 1.** Baseline County-Level Covariates Based on Tertiles of Supplemental Nutrition Assistance Program (SNAP) Policy Index Value in 2009^a^

Covariate	Median (IQR)			
	First tertile (n = 1234)	Second tertile (n = 1109)	Third tertile (n = 791)	Total (N = 3134)
SNAP policy index range^b^	0.1-3.6	3.7-5.7	5.8-9.9	0-9.9
Metropolitan counties, No. (%)^c^	375 (30.4)	423 (38.1)	367 (46.5)	1165 (37.2)
Household income, $	47 330 (41 422-54 578)	50 104 (42 509-58 555)	51 959 (42 932-62 758)	50 441 (42 415-58 555)
No. of county residents	18 802 (8701-43 522)	27 234 (11 328-67 443)	42 285 (18 639-111 252)	25 880 (11 154-66 748)
County resident characteristics, %				
Race and ethnicity				
Black, non-Hispanic	6.9 (1.9-18.5)	8.5 (4.0-17.4)	7.1 (2.9-17.8)	8.5 (2.8-18.5)
Hispanic, any race	11.9 (3.8-23.8)	12.6 (4.7-31.6)	6.6 (3.5-14.7)	9.4 (4.1-23.6)
White, non-Hispanic	66.5 (44.3-84.6)	60.1 (43.7-77.5)	73.0 (59.2-85.6)	67.1 (48.2-82.6)
Other race, non-Hispanic^d^	4.3 (2.5-7.0)	6.1 (3.2-14.4)	5.2 (3.3-7.7)	5.1 (3.0-8.1)
Age, y				
<18	25.1 (23.6-27.7)	24.1 (22.6-25.2)	23.4 (21.8-25.0)	24.0 (22.5-25.7)
19-64	62.9 (60.9-63.9)	63.1 (61.2-64.5)	62.8 (61.6-64.7)	62.9 (61.4-64.4)
≥65	11.9 (9.5-14.1)	12.0 (10.7-14.3)	13.1 (11.6-14.7)	12.3 (10.7-14.5)
Unemployed	8.2 (7.1-10.2)	10.1 (8.4-11.6)	8.8 (7.6-10.1)	9.1 (7.6-10.6)
Living in poverty	15.9 (11.2-17.9)	14.4 (11.1-16.8)	13 (9.7-16.6)	14.4 (10.7-17.1)
No health insurance (among those 18-64 y old)	21.6 (17.4-27)	22.8 (17.7-27.5)	15.5 (12.6-21.8)	20.3 (15.4-25.1)
No high school (among those ≥25 y old)	13.9 (9.9-17.5)	13.6 (11.2-19.5)	11.2 (8.9-14.4)	13 (9.9-17.1)
Living with disability	12.4 (10.3-15.1)	12.1 (10.1-15.0)	12.2 (10.2-14.7)	12.2 (10.2-14.9)
Living in single-parent households	9.6 (8.3-11.5)	10.0 (8.2-11.1)	9 (7.7-10.6)	9.6 (8.1-11.0)
Non-English speakers (among those ≥5 y old)	5.7 (2.5-12.3)	7.2 (2.8-16.7)	4.7 (2.5-9.3)	5.6 (2.6-13.0)
Living in multiunit structure	10.4 (4.5-17.0)	12.1 (5.5-19.1)	10.9 (6.0-14.5)	11.0 (5.4-17.6)
Living in mobile home	3.6 (1.8-9.9)	3.7 (1.5-9.1)	2.8 (0.7-6.7)	3.4 (1.5-8.7)
Living in crowded unit (>1 occupant per room)	2.5 (1.7-4.0)	2.7 (1.8-5.9)	1.9 (1.3-2.9)	2.4 (1.5-4.1)
No vehicle in household	6.1 (4.7-7.9)	6.8 (5.1-8.9)	7.9 (5.8-10.9)	6.9 (5.2-9.4)

^a^ Baseline county-level measures based on 2008 to 2013 5-year American Community Survey data.

^b^ The SNAP policy index is scaled from 0.1 to 10, with a higher value indicating greater adoption of policies associated with SNAP participation. SNAP policy index and adoption of individual policies were obtained from the US Department of Agriculture’s SNAP policy database.^15^

^c^ Based on 2013 US Department of Agriculture Rural-Urban Continuum Codes.

^d^ The other race category includes those reported as American Indian, Alaska Native, Asian, Native Hawaiian, Pacific Islander, or more than 1 race, and was grouped together owing to small sample sizes.

### SNAP Policy Index

**Figure 2.**
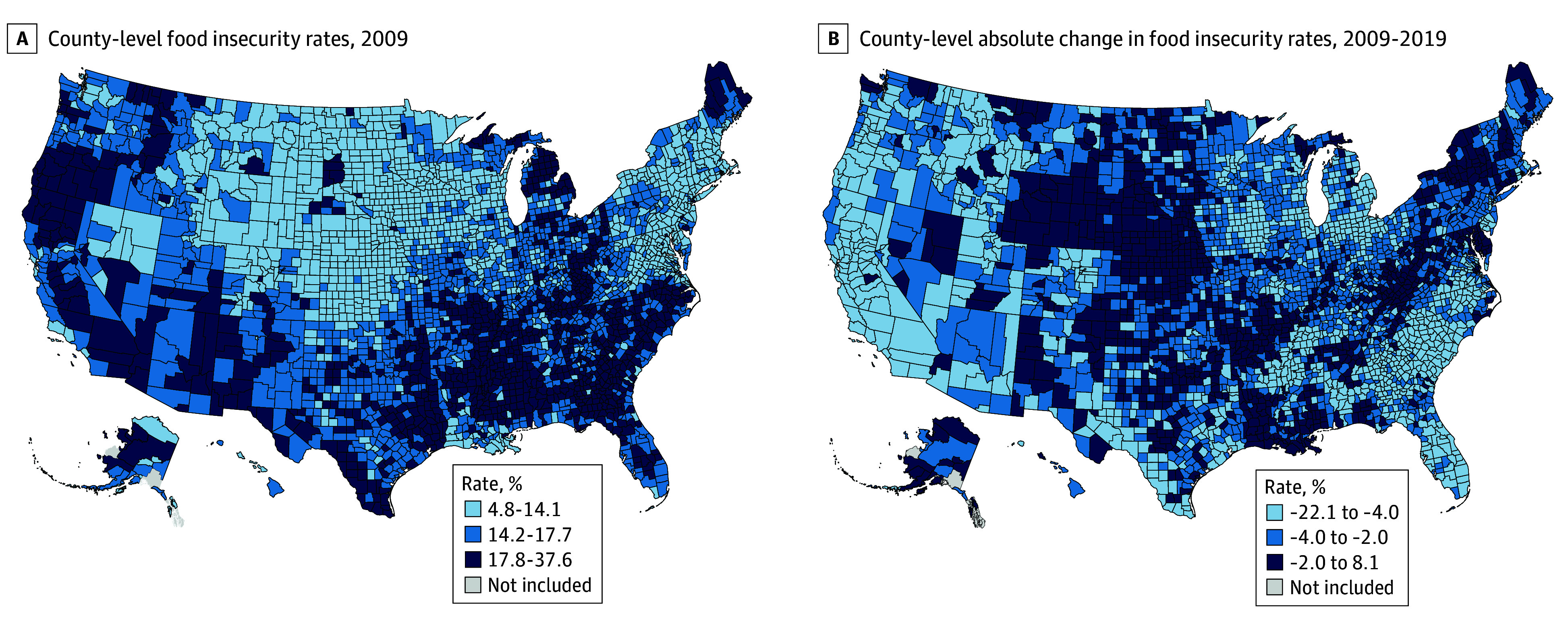
Baseline and Absolute Change in County-Level Food Insecurity Rates County food insecurity rates were obtained from the Feeding America Map the Meal Gap dataset.^17^

Between 2009 and 2019, the median (IQR) SNAP policy index across all states increased from 4.7 (3.1-6.6) to 6.8 (5.4-7.7) (eFigure 1 in Supplement 1). The median (IQR) absolute change in the index across the study period was 1.3 (0-3.0), with the greatest increase in Illinois (2.4 to 6.8) and the greatest decrease in Wisconsin (8.8 to 6.8) (Figure 1). Population-weighted median (IQR) state SNAP participation rates among individuals in low-income households increased from 34.5% (30.0%-39.9%) in 2009 to 46.5% (40.3%-50.2%) in 2013, then declined to 40.5% (35.9%-45.6%) in 2019 (eFigure 2 in the Supplement). The median (IQR) population-weighted county-level FI rates declined from 16.0% (13.2%-17.6%) to 11.2% (9.3%-13.4%) (eFigure 3 in Supplement 1). County-level baseline and absolute changes in FI rates are displayed in Figure 2.

### Marginal Structural Models

In the marginal structural model, after accounting for baseline SNAP index levels and year fixed effects, a 1-point greater increase in the SNAP policy index was associated with a 0.7–percentage point (pp; 95% CI, 0.3-1.2 pp; *P* = .002) higher state-level SNAP participation rate among residents with low income and a 0.1-pp (95% CI, 0.2-0.02 pp; *P* = .02) lower county-level FI rate (Table 2). In the models including change in each SNAP-related policy separately, instead of as a composite index, adoption of BBCE and exclusion of vehicles from asset tests were independently associated with a 1.4-pp (95% CI, 0.02-3.6 pp) and 1.4-pp (95% CI, 0.05-3.7 pp) increase in state SNAP participation rates, respectively. No individual policy was independently associated with county FI rates. However, BBCE, online application availability, simplified reporting of earnings, and vehicle exclusion from asset test were associated with a lower FI rate that was not statistically significant.

**Table 2.** Change in State Supplemental Nutrition Assistance Program (SNAP) Participation and County Food Insecurity Rates Associated With Change in SNAP Index or Policies^a^

Model variable	Change in state SNAP participation among individuals with low income, estimate (95% CI), pp^b^	*P* value	Change in county food insecurity, estimate (95% CI), pp^c^	*P* value
SNAP policy index^d^	0.7 (0.3 to 1.2)	.002	−0.1 (−0.2 to −0.02)	.02
Individual SNAP-related policies model^e^				
Broad-based categorical eligibility	1.4 (0.02 to 3.6)	.049	−0.9 (−0.2 to 0.05)	.20
Combined application project	0.2 (−0.8 to 1.4)	.82	0.01 (−0.2 to 0.2)	.99
Fingerprinting not required	0.6 (−0.7 to 1.9)	.46	0.02 (−0.4 to 0.4)	.93
Eligibility for legal noncitizen adults	1.2 (−2.3 to 5.0)	.50	0.9 (−0.2 to 1.9)	.11
Eligibility for legal noncitizen older adults	−0.5 (−4.4 to 2.4)	.80	−0.3 (−1.3 to 0.7)	.58
Online application availability	0.1 (−0.3 to 0.6)	.59	−0.1 (−0.2 to 0.03)	.18
Simplified reporting of earnings	−0.5 (−1.8 to 0.9)	.46	−0.2 (−0.5 to 0.04)	.10
Average certification period length	1.7 (−0.8 to 4.6)	.17	0.1 (−0.4 to 0.7)	.67
Vehicle exclusion from asset test	1.4 (0.05 to 3.7)	.04	−0.2 (−0.7 to 0.3)	.41

Abbreviation: pp, percentage point.

^a^ Marginal structural model includes baseline value (of SNAP index or individual policy), absolute change in value from 2009 to each individual year, and year fixed effects. Estimates shown are for the coefficient for the absolute change variable.

^b^ State SNAP participation rates among individuals living in households with income up to 200% of the federal poverty limit. State SNAP participation data were obtained from the University of Kentucky Center for Poverty Research.^12^ Data for individuals in low-income households were obtained from the annual American Community Survey.

^c^ County food insecurity rates were obtained from the Feeding America Map the Meal Gap dataset.^17^

^d^ The SNAP policy index is scaled from 0.1 to 10, with a higher value indicating greater adoption of policies associated with SNAP participation. SNAP policy index and adoption of individual policies were obtained from the US Department of Agriculture’s SNAP policy database.^15^

^e^ Single model with each policy included as covariates. The following policies were not included, as there was no variation across the study period: proportion of SNAP benefits accounted for by electronic benefit transfer and eligibility for legal noncitizen children (<18 years old).

### Simulated Trajectories

**Figure 3.**
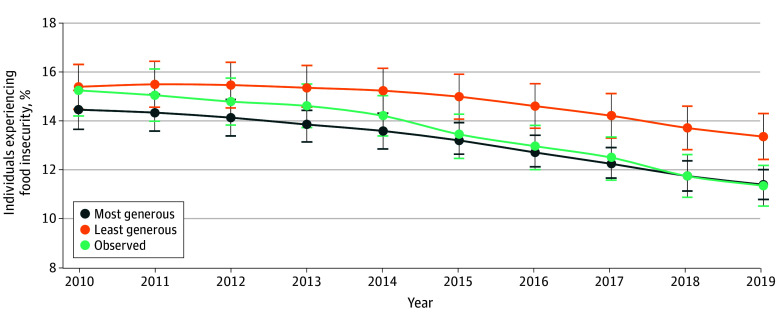
Estimated Annual Population-Weighted Food Insecurity Rates Based on Potential Supplemental Nutrition Assistance Program (SNAP) Policy Index Scenarios Least generous scenario indicates that all states adopted policies that correspond to the SNAP policy index value of the least generous state in each year. Most generous scenario indicates that all states adopted policies that correspond to a SNAP policy index value of the most generous state in each year. The annual SNAP index values used for each scenario are listed in eTable 1 in Supplement 1. All data points are annual, population-weighted, county-level food insecurity rates across all counties. Error bars indicate 95% CIs of the annual estimates. County food insecurity rates were obtained from the Feeding America Map the Meal Gap dataset.^17^ SNAP policy index and adoption of individual policies were obtained from the US Department of Agriculture’s SNAP policy database.^15^

Based on 2 simulated trajectories—having the index value of the most generous state in each year from 2009 to 2019 or having the value for the least generous state in each year—FI rates in the more generous scenario would be 2.0-pp (95% CI, 1.2-2.8 pp) lower than in the least generous scenario in 2019 (Figure 3 and eTables 3 and 4 in Supplement 1). With the 2019 US population, this translates to approximately 6.5 million (95% CI, 3.8-9.1 million) fewer individuals with FI (eTable 4 in Supplement 1). Compared to the observed trajectory of population-weighted FI rates across all US counties, FI in the most generous scenario was statistically significantly lower in the years 2010 to 2014, at −0.8 pp (95% CI, −1.3 to −0.3 pp) in 2010 to −0.6 pp (95% CI, −1.2 to −0.1 pp) in 2014, with a mean difference of −0.7 pp, followed by a convergence between the observed and simulated rates. Using the 2010 to 2014 US population, this translates to approximately 2.5 million (95% CI, 1.0-3.9 million) in 2010 to 2.0 million (95% CI, 0.3-3.7 million) in 2014, with a mean difference of 2.3 million fewer people experiencing FI. Annual FI estimates for each trajectory and differences between the trajectories are listed in eTables 3 and 4 in Supplement 1.

### Sensitivity Analysis

In the marginal structural model using state-level FI as the outcome, the model estimate was similar in magnitude and direction to the county-level FI model, with 0.1-pp (95% CI, 0.04-0.2 pp) lower state FI rate. In the household logistic regression model, among 118 824 individuals in low-income households, after accounting for household, individual, and state-level variables, a 1-point increase in the SNAP index was associated with a statistically significant lower odds of FI (odds ratio [OR], 0.97; 95% CI, 0.95-0.99) (eTable 5 in Supplement 1). In the model with each policy included individually, there were statistically significant associations between a lower FI and BBCE (OR, 0.89; 95% CI, 0.81-0.97) and combined application processing with supplemental security income (OR, 0.59; 95% CI, 0.43-0.82).

## Discussion

Between 2009 and 2019, adoption of state-level SNAP policies that reduce barriers and expand eligibility for SNAP participation were associated with higher state-level SNAP participation and lower county-level FI rates. In a simulation comparing different possible trajectories, adoption of policies that reduce barriers was associated with substantially fewer individuals experiencing FI in the US.

As one of the largest counter-cyclical programs in the US, SNAP serves as a major check on poverty during economic downturns.^25,26^ Unwinding of temporary increases in SNAP benefit amounts during the COVID-19 pandemic have been linked to increases in FI.^27^ FI rates have increased recently from 10.2% of households affected in 2021 to 13.5% in 2023.^5^ While increasing benefit amounts may be an important lever for addressing FI, this analysis suggests that policies that reduce barriers to SNAP participation and expand eligibility may also play a role in reducing FI rates. Additionally, if all states had adopted policies associated with greater participation, the decline in FI rates seen in the US between 2009 and 2019 may have been faster, and potentially millions of Americans may have avoided FI.

In this analysis we noted that after accounting for all individual policies, adoption of BBCE and exclusion of vehicles from asset tests were independently associated with statistically significant higher SNAP participation. Adoption of BBCE was also associated with a nonstatistically significant lower county FI and a statistically significant lower household-level FI rate. This finding is consistent with 2 previous studies that found that adoption of BBCE was associated with the largest magnitude of increase in SNAP participation compared to other SNAP-related policies.^8,9^ Adoption of BBCE can reduce the complexity of the SNAP application process for households. BBCE also allows states to increase the income limits under which a household qualifies for SNAP. As of 2025, 41 states and Washington, DC, have adopted BBCE.^7^ However, 9 states (Alaska, Arkansas, Kansas, Mississippi, Missouri, South Dakota, Tennessee, Utah, and Wyoming) have not adopted BBCE or have done so to a limited degree. In the 2021 to 2023 period, not accounting for differences in socioeconomic or demographic factors, 13% of all households in these states were food insecure, compared to 12% in states that had adopted BBCE.^5^ Additionally, SNAP participation rates among individuals with income lower than 200% of the FPL were 30.8% in these states in 2023 compared to 45.6% in the US overall. Therefore, it is possible that adoption of BBCE in these states could play a role in reducing FI. Other policies included in the index, such as eligibility for noncitizens, are likely to affect a subsection of the population, which may not be apparent when assessing overall county- or state-level outcomes.

### Limitations

This analysis has some limitations. Although the g-computation approach controls for measured confounding, including time-dependent confounding, it does not account for unmeasured confounders. However, the model did include a large number of county- and state-level covariates that have previously been shown to be associated with area-level health outcomes. Model-based estimates, rather than directly measured levels, for county FI were used. Given the considerable variation in economic and demographic factors within states, using an estimate of FI more granular than the state level allows for greater precision in the estimates. However, in a model with state-level covariates, we did find that the magnitude and direction of the association between change in the SNAP index and FI at the state level were similar to the county-level estimates. Additionally, the SNAP index was also associated with lower FI in a sensitivity analysis using household-level CPS data. For calculation of state SNAP participation rates, we used the number of people with household income lower than 200% of the FPL, as the true population of households potentially eligible for SNAP is unavailable. Additionally, this did not exclude certain segments of the population, such as noncitizens who may not be eligible. However, this approach has been used previously, as this population represents the segment of the population that may potentially benefit from programs such as SNAP.^9^

## Conclusions

This cross-sectional study shows that between 2009 and 2019, greater adoption of state SNAP policies aimed at reducing barriers to participation and expanding eligibility were associated with higher SNAP participation and a reduction in FI rates. Universal adoption of such policies may be associated with lower FI rates across the US.

## Notes

Supplement 1.
eMethods
**eFigure 1. **Annual median SNAP policy index values across all states **eFigure 2. **Median population-weighted state SNAP participation among households with income<200% of the Federal Poverty Limit **eFigure 3. **Annual, population-weighted, county food insecurity rates across all US counties **eTable 1. **Annual SNAP policy index values in each state **eTable 2. **Adoption of SNAP policies in 2009 by tertile of baseline SNAP policy index **eTable 3. **Estimated annual population-weighted food insecurity rates and total number of people experiencing food insecurity based on potential SNAP policy index scenarios **eTable 4. **Difference in estimated annual population-weighted food insecurity rates and total number of people experiencing food insecurity between observed values and values based on potential SNAP policy index scenarios **eTable 5.** Logistic regression model for the odds ratio of household food insecurity among individuals in low-income households associated with 1-point change in SNAP index or policies

Supplement 2.
Data Sharing Statement
