# Supplementary material for: Supplemental Nutrition Assistance Program Policies and Food Insecurity
Source: JAMA Health Forum. 2025 Dec 12;6(12):e255597. doi: 10.1001/jamahealthforum.2025.5597 (PMC12701512; doi:10.1001/jamahealthforum.2025.5597)
Supplement: Supplement 1. — eMethods eFigure 1. Annual median SNAP policy index values across all states eFigure 2. Median population-weighted state SNAP participation among households with income<200% of the Federal Poverty Limit eFigure 3. Annual, population-weighted, county food insecurity rates across all US counties eTable 1. Annual SNAP policy index values in each state eTable 2. Adoption of SNAP policies in 2009 by tertile of baseline SNAP policy index eTable 3. Estimated annual population-weighted food insecurity rates and total number of people experiencing food insecurity based on potential SNAP policy index scenarios eTable 4. Difference in estimated annual population-weighted food insecurity rates and total number of people experiencing food insecurity between observed values and values based on potential SNAP policy index scenarios eTable 5. Logistic regression model for the odds ratio of household food insecurity among individuals in low-income households associated with 1-point change in SNAP index or policies [file jamahealthforum-e255597-s001.pdf]

Supplemental Online Content

Potluri S, Venkataramani AS, Lorch SA, Illenberger N, Khatana SAM. Supplemental Nutrition Assistance Program policies and food insecurity. *JAMA Health Forum*. 2025;6(12):e255597. doi:10.1001/jamahealthforum.2025.5597

eMethods
eFigure 1. Annual median SNAP policy index values across all states
eFigure 2. Median population-weighted state SNAP participation among households with income<200% of the Federal Poverty Limit
eFigure 3. Annual, population-weighted, county food insecurity rates across all US counties
eTable 1. Annual SNAP policy index values in each state
eTable 2. Adoption of SNAP policies in 2009 by tertile of baseline SNAP policy index
eTable 3. Estimated annual population-weighted food insecurity rates and total number of people experiencing food insecurity based on potential SNAP policy index scenarios
eTable 4. Difference in estimated annual population-weighted food insecurity rates and total number of people experiencing food insecurity between observed values and values based on potential SNAP policy index scenarios
eTable 5. Logistic regression model for the odds ratio of household food insecurity among individuals in low-income households associated with 1-point change in SNAP index or policies

This supplemental material has been provided by the authors to give readers additional information about their work.

eMethods

SNAP policy index:

The following policies, that have previously been demonstrated to be associated with SNAP participation rates, were used to construct the index:

Broad-based categorical eligibility (BBCE): Indicator for whether the state uses BBCE to increase or eliminate the asset test and/or to increase the gross income limit for all or most SNAP applicants. Households that receive cash assistance through programs such as Temporary Assistance for Needy Families (TANF) and supplemental security income (SSI) typically also qualify for SNAP. The Personal Responsibility and Work Opportunity Reconciliation Act of 1996 that replaced the previous, cash-assistance based, Aid to Families with Dependent Children (AFDC) program with TANF also established BBCE as an option for states to extend SNAP benefits to households that qualify for non-cash assistance through TANF (e.g., childcare subsidies) or state maintenance of effort (MOE) funded benefits.

Exemption of vehicles from asset tests: Variable indicating whether the state excludes vehicles in the household from the SNAP asset test. If all vehicles were excluded, a value of 1 was given, if only one vehicle was excluded, then a value of 0.5 was given.

Average recertification period: The average certification period (in months) for households receiving SNAP. The monthly mean value for the average recertification period across the following types of households was calculated: those with earnings, those without earnings and no senior members, and those without earnings and with at least one senior (age 60+) member. Then the mean annual was calculated and rescaled to be between 0 and 1, with higher values indicating a longer recertification period (which indicates fewer barriers for SNAP participation).

© 2025 Potluri S et al. *JAMA Health Forum*.

Eligibility for legal non-citizens: Variable indicating whether legal noncitizen who satisfy other SNAP eligibility requirements are considered to be eligible for Federal SNAP benefits or State-funded food assistance. If all residents were eligible, then a value of 1 was given and if some, but not all, residents were considered eligible, then a value of 0.5 was given. Separate variables for non-elderly adults (18 to 64 years old), elderly (65 and older) and children (under 18 years of age) were created.

Combined application with SSI: Indicator for whether the state operates a Combined Application Project for SSI recipients that allows SSI recipients to use a streamlined SNAP application process.

Fingerprinting requirements: Variable indicating whether a state requires fingerprinting of SNAP applicants. A value of 0 was used if fingerprinting was required statewide, a value of 0.5 if it was required in some parts of the state, and 1 if no fingerprinting was required.

Online application availability: Indicator for whether a state allows households to submit a SNAP application online. A value of 0 was used if online application was not available, a value of 0.5 if it was available in some parts of the state, and 1 if it was available throughout the state.

EBT (electronic benefit transfer) issuance: Monthly proportion of all SNAP benefits that are issued through EBT. The mean annual value was then calculated for each state.

Simplified reporting of earnings: Indicator for whether a state uses the simplified reporting option for households with earnings. This reduces requirements for reporting changes in household circumstances.

For policies that were implemented for less than 12 months in a calendar year, the fraction of months in which the policy was implemented was used.

© 2025 Potluri S et al. *JAMA Health Forum*.

After creating each of the covariates listed above, the unweighted mean value was calculated and then scaled to be between 0.1 and 10. A higher value indicates adoption of more policies that reduce barriers to SNAP participation or increase eligibility.

Components of the Social Vulnerability Index:

County and state-level covariates used to construct the 2014 Centers for Disease Control and Prevention's Social Vulnerability Index (SVI) were obtained from the US Census Bureau. The following covariates compose the SVI:

Proportion of residents with income below poverty level

Proportion of residents unemployed

Per capita income

Proportion of residents without high school diploma

Proportion of residents who are 65 years of age or older

Proportion of residents who are 17 years of age or younger

Proportion of civilian residents with a disability

Proportion of households that are single-parent households

Proportion of residents who are not non-Hispanic white

Proportion of residents who speak English "less than well"

Proportion of housing units that are in multi-unit structures

Proportion of housing units that are mobile homes

Proportion of housing units with more people than rooms

Proportion of households with no vehicle

Proportion of the population living in group quarters

© 2025 Potluri S et al. *JAMA Health Forum*.

For this analysis, we used values for the proportion of residents who were non-Hispanic Black and the proportion of residents were Hispanic (any race), instead of the proportion of non-white residents. We also used the median household income (inflation adjusted to 2009) instead of the per-capita income.

Annual values of the median household income, percentage of residents who were non-Hispanic Black or Hispanic, percentage of residents 17 and younger and percentage 65 and older, percentage of residents living in poverty, and percentage unemployed were available at the county level over the study period. For the remainder of the SVI components, for the county-level models, data from the 5-year ACS was used (2008-2013 for years 2009 to 2013, 2014-2018 for 2014, and 2015-2019 for years 2015 to 2019). For the state-level models data from 1-year ACS was used.

Longitudinal g-computation

Introduction and Assumptions:

G-computation is a robust approach for estimating the causal effect of a time-varying intervention in the presence of time-varying confounding.[18] This approach is particularly useful in situations where there is "feedback loop" confounding (state characteristics influence state policies, which influence state characteristics, which influence state policies…). To isolate the causal effect of interest, this procedure relies on the following identification assumptions:

© 2025 Potluri S et al. *JAMA Health Forum*.

1. (*Sequential Ignorable Treatment Assignment*): At any time, t, the distribution of potential outcomes is independent of the observed treatment assignment conditional on measured covariates:
    a. $Y_t(\bar{s}_t) \perp \bar{S}_t | \bar{X}_t, \bar{S}_{t-1}, \bar{Y}_{t-1}$
2. (*Positivity*) For all possible covariate histories at time t (those with positive probability), the probability of all possible exposure levels is non-zero.
    a. If $\Pr(\bar{X}_t, \bar{S}_{t-1}, \bar{Y}_{t-1}) > 0$, then $\Pr(S_t = s_t | \bar{X}_t, \bar{S}_{t-1}, \bar{Y}_{t-1}) > 0$ for all s_t
3. (*Consistency*) Observed outcomes at time t are equivalent to the corresponding potential outcome under the observed treatment.
    a. $Y_t = Y_t(\bar{s}_t)$ for all t

Overview of approach:

Let $X_t(\bar{s}_t)$ denote the value of the random variable X_t that would be observed if we were to intervene to set SNAP policy indices up to time t as $\bar{s}_t$ (where we use overbar notation to denote $\bar{S}_t = (S_{2009}, S_{2010}, \ldots, S_t)$). If the identification assumptions of no unmeasured confounding, positivity, and SUTVA hold, then g-computation provides a procedure for drawing data from the joint distribution of potential outcomes for any pre-defined trajectory of SNAP indices up to the year 2019, $\bar{s}_{2019}$:

$$D(\bar{s}_{2019}) = (X_{2009}(\bar{s}_{2009}), Y_{2009}(\bar{s}_{2009}), \ldots, X_{2019}(\bar{s}_{2019}), Y_{2019}(\bar{s}_{2019}))$$

We describe the process of sampling from this distribution in more detail in the following section. If we pre-select a large number of potential trajectories ($\bar{s}_{2019,k}$ for $k = 1, \ldots m$), and draw a sample from the joint distribution of potential outcomes under each trajectory ($D(\bar{s}_{2019,k})$ for $k = 1, \ldots, m$), then we can regress our potential outcomes on our predictors to estimate the

© 2025 Potluri S et al. *JAMA Health Forum*.

parameters in a marginal structural model. Specifically, because we are interested in the effect of changes in SNAP policies on Food Insecurity we fit the model:

$$(1)\ Y_{ijt} = \beta_0 + \beta_1 S_{i,2009} + \beta_2 \left(S_{i,t} - S_{i,2009}\right) + \beta_T T + \gamma_i + e_{ijt}$$

Here, we regress Food Insecurity rates in county j of state i during year t on state-level SNAP policies in the year 2009/baseline ($S_{i,2009}$), the change in SNAP policies from 2009 and year t in state i, year-level fixed effects (summarized as T), and a random state-level intercept (γ_i).

As an alternative to the marginal structural model approach, we may prespecify a treatment rule (e.g. Set all SNAP policy indices to 8 throughout the course of observation) and draw from the distribution of potential outcomes under this treatment rule. Given a sample from this distribution, we may then estimate the mean Food Insecurity rate under this selected intervention. In our study, we estimated the difference in state-level SNAP participation and county-level food insecurity rates across the US from 2009 to 2019 under two hypothetical scenarios – all states adopting the most generous state policies for each year compared to all states adopting the least generous policies for each year.

Sampling from the Joint Distribution of potential outcomes:

The mechanism for sampling from this distribution relies on (1) an estimation component, and (2) a sampling component. In the estimation component, we use flexible modelling approaches to estimate the relationship between the confounding, exposure, and outcome variables. In the sampling component, we use estimates of the joint distribution of data to simulate what would have occurred under an intervention on SNAP policies. Sampled data can then be used to determine how counterfactual interventions on SNAP policies may have influenced Food Insecurity rates.

© 2025 Potluri S et al. *JAMA Health Forum*.

In the context of our problem, the estimation component consists of fitting appropriate models for the following set of distributions:

- The distribution of Y_{2009} conditional on S_{2009} and X_{2009}
  - Denoted $f(Y_{2009}|S_{2009}, X_{2009})$
- The distribution of Y_t conditional on $S_t, S_{t-1}, X_t, X_{t-1}$, and Y_{t-1} for $t = 2010, \dots, 2019$
  - Denoted $f(Y_t|S_t, X_t, S_{t-1}, X_{t-1}, Y_{t-1})$
- The distribution of X_t conditional on Y_{t-1}, S_{t-1}, and X_{t-1} for $t = 2010, \dots, 2019$
  - Denoted $f(X_t|S_{t-1}, X_{t-1}, Y_{t-1})$

Due to the large number of predictors and observations, we used a random forest algorithm to estimate each of the selected distributions. Given estimates of the mentioned components ($\hat{f}(Y_{2009}|S_{2009}, X_{2009}), \hat{f}(X_t|S_{t-1}, X_{t-1}, Y_{t-1})$, and $\hat{f}(Y_t|S_t, X_t, S_{t-1}, X_{t-1}, Y_{t-1})$), and a prespecified treatment pattern ($\bar{s}_{2019,k}$), then we can sample from the joint distribution of potential outcomes as follows:

1. Select a large number of desired sampling units, m.
2. Draw m observations from $\hat{f}(X_{2009})$, X^*_{2009}. (This is obtained from the empirical distribution and does not need to be estimated)
3. Use the sampled X^*_{2009} and pre-specified s_{2009}, to draw m observations from $\hat{f}(Y_{2009}|s_{2009}, X^*_{2009})$, Y^*_{2009}.
4. Starting at $t = 2010$ until 2019, sequentially draw m samples from:
   a. $\hat{f}(X_t|s_{t-1}, X^*_{t-1}, Y^*_{t-1})$, X^*_t
   b. $\hat{f}(Y_t|s^*_t, X^*_t, s^*_{t-1}, X^*_{t-1}, Y^*_{t-1})$, Y^*_t
   c. Set $t == t + 1$
5. Compile the sample $((X^*_{2009}(\bar{s}_{2009}), Y^*_{2009}(\bar{s}_{2009}), \dots, X^*_{2019}(\bar{s}_{2019}), Y^*_{2019}(\bar{s}_{2019}))$

© 2025 Potluri S et al. *JAMA Health Forum*.

Logistic regression model

Data from the annual food security supplement of the Current Population Survey for the years 2009-2019 was used to fit logistic regression models with the odds of experiencing food insecurity as the outcome. A single observation for each household was used in each year. Individual level covariates for the head of the household were also included in the models as well as year and state fixed effects and state level covariates as included in the g-computation models. Person-level weights and robust standard errors clustered at the state-level were used for analysis.

The following variables were included in the model:

State fixed effects

Year fixed effects

Age of head of household

Sex of head of household

Educational attainment of head of household (less than high school, high school graduation, at least some college, at least some post-graduate education)

Race of head of household (white, black, Asian, American Indian/Alaska Native, Hawaiian/Pacific Islander, more than one race)

Hispanic Ethnicity (Not Hispanic, Mexican, Puerto Rican, Cuban, Dominican, Salvadoran, Central/South American, Central American, South American, Other Hispanic)

Martial status of head of household (Married – spouse present, Married – spouse absent, Separated, Divorced, Widowed, Never Married/Single)

Indicator for labor force participation for head of household

© 2025 Potluri S et al. *JAMA Health Forum*.

Employment status for head of household (at work, has job – not at work last week, unemployed – experienced worker, unemployed – new worker, unable to work, retired, other)

Indicator for immigrant status for head of household

Indicator for non-US citizenship for head of household

Hearing difficulty for head of household

Vision difficulty for head of household

Memory difficulty for head of household

Physical difficulty for head of household

Mobility difficulty for head of household

Personal care limitation for head of household

Type of household (husband/wife primary family – neither in armed forces, husband/wife primary family – either/both in armed forces, unmarried civilian male – primary family householder, unmarried civilian female – primary family householder, civilian male primary individual, civilian female primary individual, group quarters with family, group quarters without family)

Number of members in household

Number of children in household

Core based statistical area size (Non-metropolitan, 100,000-249,999, 250,000-499,999, 500,000-999,999, 1,000,000-2,499,999, 2,500,000-4,999,999, 5,000,000 or more)

All state-level level variables used in g-computation models also included based on household state of resident

© 2025 Potluri S et al. *JAMA Health Forum*.

All continuous variables were included as linear splines with 3 equally sized groups. Baseline SNAP index values (from 2009) and the absolute change in the SNAP index from baseline were also included in the model. For the model with individual policies, the baseline and absolute change in each policy were included.

© 2025 Potluri S et al. *JAMA Health Forum*.

eFigure 1 – Annual median SNAP policy index values across all states

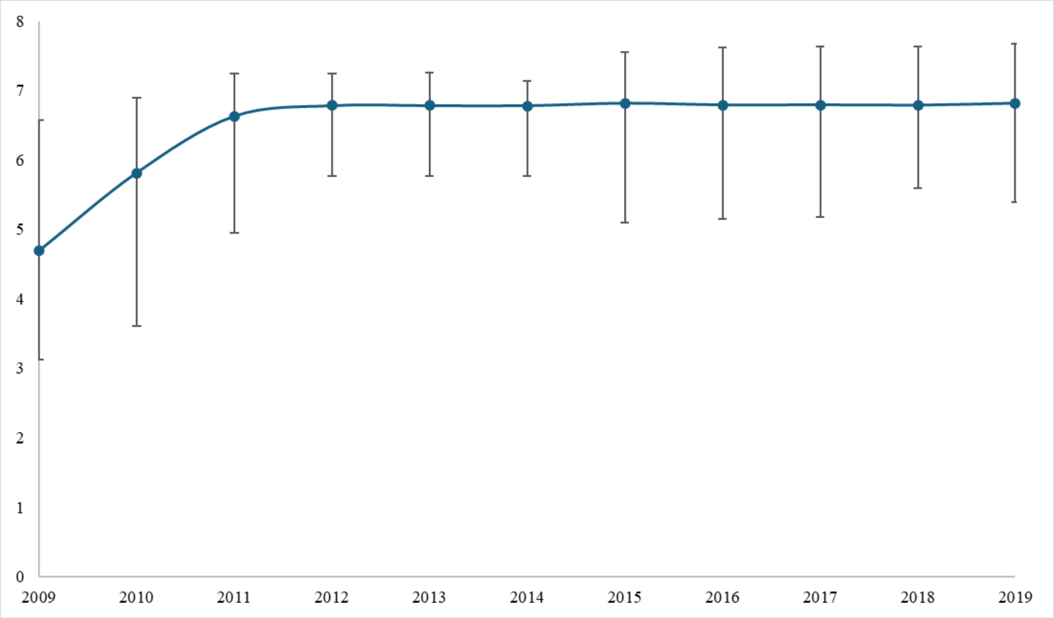

The SNAP policy index is scaled from 0.1 to 10, with a higher value indicating greater adoption of policies associated with SNAP participation. SNAP policy index and adoption of individual policies obtained from the US Department of Agriculture's SNAP policy database
Error bars indicate interquartile range

© 2025 Potluri S et al. *JAMA Health Forum*.

eFigure 2 – Median population-weighted state SNAP participation among households with income<200% of the Federal Poverty Limit

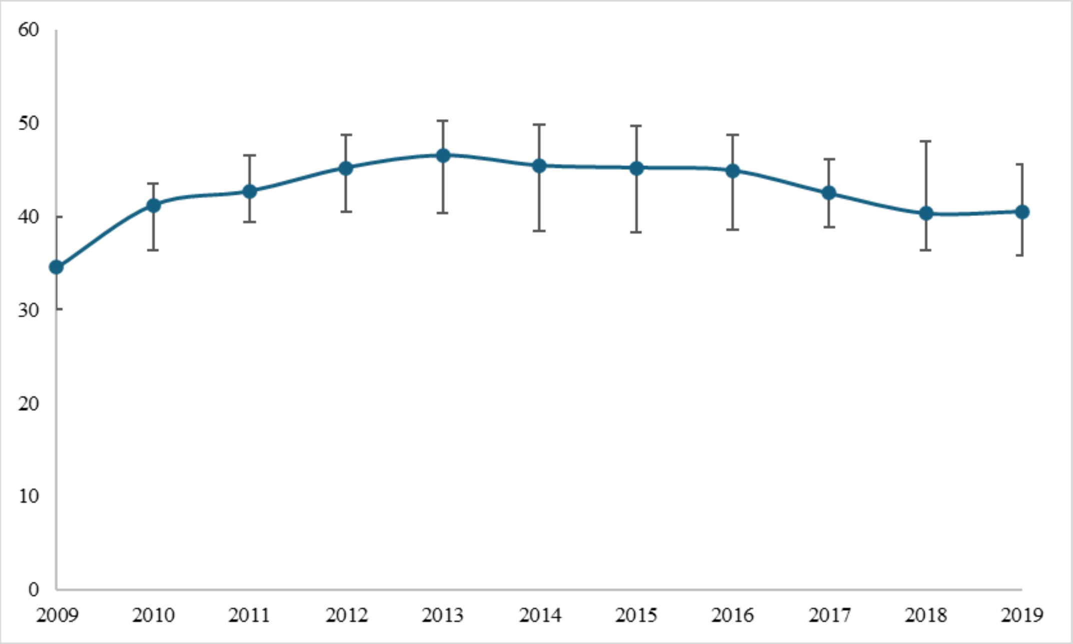

State SNAP participation rates among individuals living in households with income up to 200% of the Federal Poverty Limit. State SNAP participation data obtained from the University of Kentucky Center for Poverty Research. Individuals in low-income households obtained from the annual American Community Survey data. Data for February 2019 excluded due to federal government shutdown. Values weighted by annual state low-income population.
Error bars indicate interquartile range

© 2025 Potluri S et al. *JAMA Health Forum*.

eFigure 3 – Annual, population-weighted, county food insecurity rates across all US counties

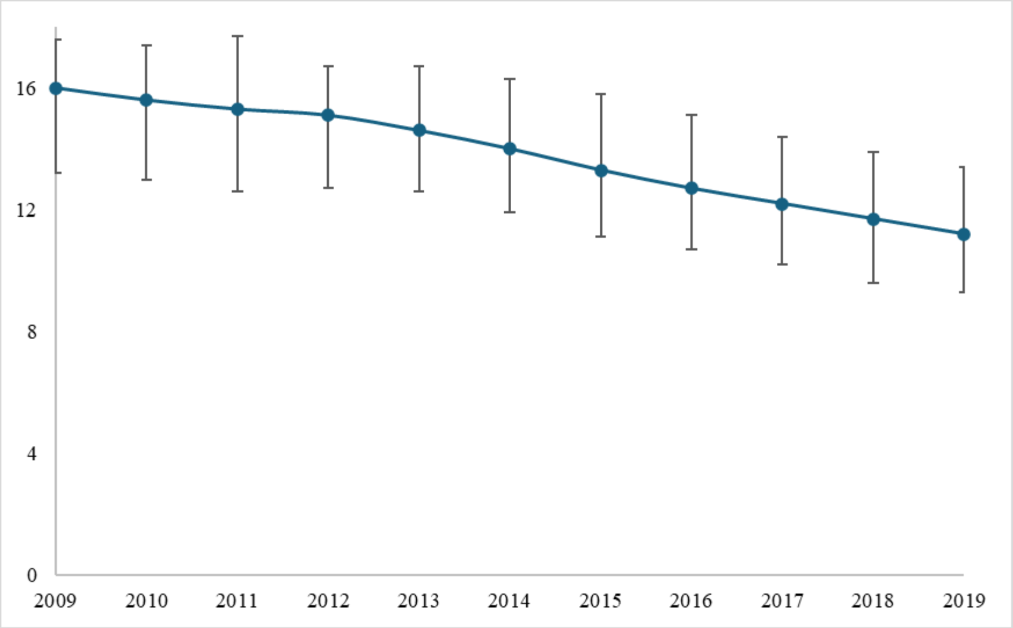

County food insecurity rates obtained from the Feeding America Map the Meal Gap dataset.
Values weighted by annual county population.
Error bars indicate interquartile range

© 2025 Potluri S et al. *JAMA Health Forum*.

eTable 1 – Annual SNAP policy index values in each state [a]

	2009	2010	2011	2012	2013	2014	2015	2016	2017	2018	2019
Alabama	2.9	5.7	6.0	6.6	7.1	7.1	7.1	7.1	7.1	7.1	7.1
Alaska	1.1	1.1	1.1	1.1 [b]	1.1 [b]	1.1 [b]	1.1 [b]	1.1 [b]	1.1 [b]	1.2 [b]	1.1 [b]
Arizona	6.5	6.8	6.8	6.8	6.8	6.8	6.8	6.8	7.1	7.8	7.8
Arkansas	1.5	1.5	2.3	2.5	2.7	2.8	2.8	2.8	2.8	2.8	2.8
California	4.8	6.3	6.4	7.7	8.1	8.9	8.9	8.9	8.9	9.0	9.1
Colorado	2.8	2.8	5.5	6.8	6.8	6.8	6.8	6.8	6.9	6.9	6.8
Connecticut	6.4	7.8	7.9	8.0	8.0	8.0	9.0	9.1	9.1	9.1	9.1
Delaware	6.6	6.7	7.0	7.0	7.0	7.0	7.0	7.0	7.0	7.0	7.0
District of Columbia	3.0	5.2	6.0	6.0	6.0	6.0	6.0	6.0	6.0	6.0	6.0
Florida	4.8	6.2	7.7	7.7	7.6	7.6	7.7	7.8	7.6	7.6	7.6
Georgia	6.6	6.6	6.6	6.6	6.6	6.6	6.6	5.6	5.6	5.6	5.6
Hawaii	2.8	3.5	5.8	5.8	5.8	5.8	5.8	5.8	5.8	5.8	5.8
Idaho	4.0	5.8	4.9	4.4	4.3	4.3	4.3	4.2	4.2	4.3	4.3
Illinois	2.4	6.0	6.8	6.8	6.9	6.9	6.8	6.8	6.8	6.8	6.8
Indiana	3.1	3.2	3.2	3.3	3.3	3.3	3.3	3.3	3.3	6.8	6.8
Iowa	2.2	2.1	6.6	6.6	6.6	6.6	6.6	6.6	6.6	6.6	6.6
Kansas	3.9	3.9	3.9	3.9	3.9	3.9	3.2	2.4	2.6	2.6	2.6
Kentucky	3.8	5.5	6.7	6.8	6.9	6.8	7.6	7.7	8.0	8.0	7.9
Louisiana	4.1	6.4	8.0	8.1	8.1	6.9	5.1	5.1	5.2	5.1	5.1
Maine	7.8	7.8	7.4	7.8	7.8	7.8	7.8	6.8	6.8	6.8	7.4
Maryland	6.6	7.1	7.6	7.7	7.6	7.6	7.7	7.7	7.7	7.8	7.8
Massachusetts	7.9	8.0	8.0	8.1	8.1	8.1	8.1	8.1	8.1	8.1	8.2
Michigan	7.3	8.0	7.2	6.5	6.5	6.5	6.5	6.5	6.5	6.5	6.7
Minnesota	6.6	6.8	6.8	7.4	7.8	7.8	7.9	7.9	7.9	7.9	7.9
Mississippi	3.8	5.7	7.1	7.1	7.3	7.3	7.4	7.5	7.3	6.2	5.4
Missouri	2.9	2.9	3.1	3.7	4.1	4.1	4.1	4.1	4.1	4.1	4.1
Montana	5.5	6.0	6.8	7.0	7.0	7.0	7.0	7.0	7.0	7.0	7.0
Nebraska	2.8	3.0	3.5	7.0	7.0	6.9	6.9	6.9	6.7	6.6	6.6
Nevada	4.5	5.6	5.7	6.6	6.6	6.6	6.6	6.6	6.6	6.6	6.6
New Hampshire	4.0	5.5	5.7	6.5	6.5	6.5	6.5	6.5	6.5	6.5	6.5
New Jersey	4.7	7.2	8.0	8.0	8.0	8.0	8.0	8.0	8.0	8.0	8.0
New Mexico	3.4	6.3	7.0	7.1	7.1	6.2	7.0	7.0	7.0	7.0	7.0
New York	7.0	7.2	7.3	8.0	8.2	8.2	8.2	8.2	8.2	8.2	8.2

© 2025 Potluri S et al. *JAMA Health Forum*.

North Carolina	3.7	5.2	6.6	6.6	6.7	6.7	7.6	7.6	7.6	7.7	7.7
North Dakota	5.7	5.6	6.4	6.6	6.6	6.6	6.6	6.6	6.6	6.6	6.6
Ohio	5.6	5.8	6.8	6.8	6.8	6.8	6.8	6.8	6.8	6.9	7.0
Oklahoma	4.8	6.0	6.0	6.0	6.0	6.0	7.0	7.0	7.0	7.0	7.0
Oregon	5.8	5.8	5.8	6.4	6.8	6.8	6.8	6.8	6.8	6.8	6.8
Pennsylvania	8.1	8.1	8.1	7.2	6.6	6.6	7.6	8.1	8.2	8.3	8.3
Rhode Island	6.1	7.0	7.0	7.0	7.0	7.0	7.0	7.1	7.1	7.0	7.1
South Carolina	6.8	7.9	7.7	7.6	7.6	7.6	7.6	7.7	7.6	7.6	7.7
South Dakota	1.0	2.4	2.4	2.4	2.4	2.4	3.3	3.4	3.4	3.4	3.4
Tennessee	3.6	3.6	3.6	3.8	3.8	3.8	3.8	3.8	3.8	3.8	3.8
Texas	3.5	3.5	4.2	4.9	4.9	4.9	4.9	4.9	4.9	4.9	4.9
Utah	3.6	3.6	3.6	3.6	3.6	3.6	3.6	3.6	3.6	3.6	3.6
Vermont	5.7	6.0	6.8	6.8	6.9	6.9	7.0	7.0	7.0	7.0	7.1
Virginia	4.9	5.0	5.0	5.0	5.0	4.9	5.0	5.0	5.0	5.1	5.0
Washington	9.9 [c]	9.9 [c]	9.9 [c]	10.0 [c]	10.0 [c]	10.0 [c]	10.0 [c]	10.0 [c]	10.0 [c]	10.0 [c]	10.0 [c]
West Virginia	6.9	6.9	6.9	6.9	6.9	6.9	7.1	7.1	7.1	7.1	7.1
Wisconsin	8.8	8.8	7.8	6.8	6.8	6.8	6.8	6.8	6.8	6.8	6.8
Wyoming	0.1 [b]	0.2 [b]	0.6 [b]	2.2	2.8	2.9	2.9	2.8	2.6	2.6	2.6

a. The SNAP policy index is scaled from 0.1 to 10, with a higher value indicating greater adoption of policies associated with SNAP participation. SNAP policy index and adoption of individual policies obtained from the US Department of Agriculture's SNAP policy database
b. Value used to create the trajectory of the least generous scenario that all states adopted policies that correspond to the lowest SNAP policy index implemented in each year.
c. Value used to create the trajectory of the most generous scenario that all states adopted policies that correspond to the highest SNAP policy index implemented in each year.

© 2025 Potluri S et al. *JAMA Health Forum*.

eTable 2 – Adoption of SNAP policies in 2009 by tertile of baseline SNAP policy index [a,b]

	**Tertile 1 (N=17 states)**	**Tertile 2 (N=17 states)**	**Tertile 3 (N=17 states)**
Range of SNAP policy index in 2009	0.1 - 3.6	3.7 - 5.7	5.8 - 9.9
	**Median (IQR)**		
State-wide average certification period length (in months)	12.4 (9.0 – 14.0)	12.1 (10.6 – 13.1)	13.3 (11.8 – 15.0)
	**Proportion with policy enacted (%)**		
Broad-based categorical eligibility (BBCE)	5.9%	52.9%	100.0%
Vehicle exclusion from asset test	One vehicle – 35.3%<br>All vehicles – 52.9%	One vehicle – 0%<br>All vehicles – 100%	One vehicle – 0%<br>All vehicles – 100%
Combined application project	17.7%	41.2%	41.2%
Fingerprinting not required	Part of state – 0%<br>Entire state – 94.1%	Part of state – 0%<br>Entire state – 94.1%	Part of state – 5.9%<br>Entire state – 88.2%
Eligibility for legal noncitizen adults	Some individuals – 94.1%<br>All individuals – 5.9%	Some individuals – 94.1%<br>All individuals – 5.9%	Some individuals – 76.5%<br>All individuals – 23.5%
Eligibility for legal noncitizen children	All individuals – 100.0%	All individuals – 100.0%	All individuals – 100.0%
Eligibility for legal noncitizen elderly adults	Some individuals – 94.1%<br>All individuals – 5.9%	Some individuals – 94.1%<br>All individuals – 5.9%	Some individuals – 70.6%<br>All individuals – 29.4%
Online application availability	Part of state – 11.8%<br>Entire state – 29.4%	Part of state – 5.9%<br>Entire state – 23.5%	Part of state – 11.8%<br>Entire state – 29.4%
Simplified reporting of earnings	94.1%	94.1%	100.0%
Electronic benefit transfer issuance	100.0%	100.0%	100.0%

a. Represents adoption of policies in December 2009
b. SNAP policy data obtained from the US Department of Agriculture's SNAP policy database

© 2025 Potluri S et al. *JAMA Health Forum*.

eTable 3 – Estimated annual population-weighted food insecurity rates and total number of people experiencing food insecurity based on potential SNAP policy index scenarios [a,b,c]

	Least generous[d]		Most generous[e]		Observed[f]	
Year	Proportion (95% CI), pp	Total (95% CI)	Proportion (95% CI), pp	Total (95% CI)	Proportion (95% CI), pp	Total (95% CI)
2010	15.4 (14.5, 16.3)	47581309 (44738041, 50424577)	14.4 (13.6, 15.2)	44666114 (42190944, 47141284)	15.2 (14.2, 16.297)	47136980 (43862994, 50410966)
2011	15.5 (14.5, 16.4)	48231018 (45289844, 51172191)	14.3 (13.6, 15.1)	44609227 (42240099, 46978356)	15.0 (14.0, 16.1)	46855888 (43494274, 50217501)
2012	15. 5 (14.5, 16.4)	48486173 (45531111, 51441235)	14.1 (13.4, 14.9)	44283157 (41954047, 46612267)	14.8 (13.8, 15.7)	46378827 (43355034, 49402619)
2013	15.3 (14.4, 16.3)	48474765 (45563605, 51385925)	13.8 (13.1, 14.5)	43709152 (41447806, 45970498)	14.6 (13.7, 15.5)	46132887 (43302358, 48963415)
2014	15.2 (14.3, 16.1)	48445020 (45493090, 51396950)	13.6 (12.8, 14.3)	43180110 (40858537, 45501682)	14.2 (13.4, 15.0)	45178527 (42539080, 47817973)
2015	15.0 (14.0, 15.9)	48007141 (45038513, 50975768)	13.2 (12.4, 13.9)	42252325 (39901966, 44602684)	13.4 (12.6, 14.3)	43077291 (40432497, 45722085)
2016	14.6 (13.7, 15.5)	47118555 (44168653, 50068457)	12.7 (12.0, 13.4)	40970140 (38678108, 43262172)	12.9 (12.1, 13.8)	41809518 (39059635, 44559401)
2017	14.2 (13.3, 15.1)	46122266 (43151156, 49093376)	12.2 (11.5, 12.9)	39712264 (37523537, 41900992)	12.5 (11.6, 13.3)	40548805 (37818972, 43278638)
2018	13.7 (12.8, 14.6)	44734204 (41818060, 47650349)	11.7 (11.1, 12.3)	38299161 (36277618, 40320704)	11.7 (10.8, 12.6)	38271612 (35403757, 41139467)
2019	13.3 (12.4, 14.3)	43770354 (40676509, 46864200)	11.4 (10.8, 12.0)	37301676 (35289674, 39313677)	11.3 (10.5, 12.2)	37156678 (34417837, 39895519)

a. SNAP index values used for each trajectory are displayed in Supplemental Table 1.
b. Total estimated number of people experiencing food insecurity based on total annual US population.
c. Food insecurity rates are annual, population-weighted, county-level food insecurity rates, across all counties. County food insecurity rates from the Feeding America Map the Meal Gap dataset.
d. Least generous scenario indicates that all states adopted policies that correspond to the SNAP index values of the least generous state in each year.
e. Most generous scenario indicates that all states adopted policies that correspond to the SNAP index values of the most generous state in each year.
f. Observed population weighted food insecurity rates

© 2025 Potluri S et al. *JAMA Health Forum*.

eTable 4 – Difference in estimated annual population-weighted food insecurity rates and total number of people experiencing food insecurity between observed values and values based on potential SNAP policy index scenarios [a,b,c]

	Difference between most generous SNAP policy trajectory and least generous SNAP policy trajectory [d,e]		Difference between most generous SNAP policy trajectory and observed values [e,f]		Difference between least generous SNAP policy trajectory and observed values [d,f]	
Year	Difference in food insecurity proportion, pp (95% CI)	Estimated absolute difference in the number of people experiencing food insecurity (95% CI)	Difference in food insecurity proportion pp (95% CI)	Estimated absolute difference in the number of people experiencing food insecurity (95% CI)	Difference in food insecurity proportion pp (95% CI)	Estimated absolute difference in the number of people experiencing food insecurity (95% CI)
2010	-0.9 (-1.5, -0.4)	-2915195 (-4567740, -1262651)	-0.8 (-1.3, -0.3)	-2470866 (-3908809, -1032922)	0.1 (-0.2, 0.5)	444330 (-573368, 1462028)
2011	-1.2 (-1.8, -0.6)	-3621790 (-5466238, -1777343)	-0.7 (-1.3, -0.2)	-2246660 (-3961739, -531582)	0.4 (0.1, 0.8)	1375130 (219380, 2530880)
2012	-1.3 (-2.0, -0.7)	-4203016 (-6238199, -2167833)	-0.7 (-1.2, -0.1)	-2095670 (-3772983, -418357)	0.7 (0.4, 1.0)	2107346 (1188049, 3026643)
2013	-1.5 (-2.2, -0.8)	-4765613 (-7008852, -2522375)	-0.8 (-1.3, -0.2)	-2423735 (-4133827, -713643)	0.7 (0.4, 1.1)	2341878 (1322038, 3361719)
2014	-1.7 (-2.4, -0.9)	-5264910 (-7695977, -2833843)	-0.6 (-1.2, -0.1)	-1998417 (-3707878, -288956)	1.0 (0.7, 1.4)	3266494 (2135390, 4397598)
2015	-1.8 (-2.6, -1.0)	-5754816 (-8344370, -3165262)	-0.3 (-0.8, 0.3)	-824966 (-2508961, 859029)	1.5 (1.1, 1.9)	4929850 (3618607, 6241092)
2016	-1.9 (-2.7, -1.1)	-6148415 (-8799672, -3497158)	-0.3 (-0.8, 0.3)	-839378 (-2618558, 939802)	1.6 (1.2, 2.1)	5309037 (3860140, 6757933)
2017	-2.0 (-2.8, -1.2)	-6410002 (-9056504, -3763500)	-0.3 (-0.8, 0.3)	-836541 (-2666154, 993072)	1.7 (1.2, 2.2)	5573461 (4028193, 7118729)
2018	-2.0 (-2.7, -1.2)	-6435043 (-8981779, -3888308)	0.01 (-0.7, 0.7)	27549 (-2189596, 2244693)	2.0 (1.4, 2.6)	6462592 (4549363, 8375821)
2019	-2.0 (-2.8, -1.2)	-6468679 (-9098017, -3839341)	0.04 (-0.6, 0.7)	144998 (-1903180, 2193176)	2.0 (1.4, 2.6)	6613677 (4714661, 8512692)

a. SNAP index values used for each trajectory are displayed in Supplemental Table 1.
b. Total estimated number of people experiencing food insecurity based on total annual US population.
c. Food insecurity rates are annual, population-weighted, county-level food insecurity rates, across all counties. County food insecurity rates from the Feeding America Map the Meal Gap dataset.
d. Least generous scenario indicates that all states adopted policies that correspond to the SNAP index values of the least generous state in each year.
e. Most generous scenario indicates that all states adopted policies that correspond to the SNAP index values of the most generous state in each year.
f. Observed population weighted food insecurity rates

© 2025 Potluri S et al. *JAMA Health Forum*.

eTable 5 – Logistic regression model for the odds ratio of household food insecurity among individuals in low-income households associated with 1-point change in SNAP index or policies[a]

Model Variable	Odds ratio (95% CI)	p-value
SNAP policy index [b]	0.97 (0.95, 0.99)	0.002
**Individual SNAP related policies model [c]**		
Broad-based categorical eligibility (BBCE)	0.89 (0.81, 0.97)	0.01
Combined application project	0.59 (0.43, 0.82)	0.001
Fingerprinting not required	0.97 (0.88, 1.07)	0.50
Eligibility for legal noncitizen adults	0.77 (0.44, 1.36)	0.37
Eligibility for legal noncitizen seniors	0.75 (0.41, 1.36)	0.35
Online application availability	1.03 (0.96, 1.12)	0.32
Simplified reporting of earnings	0.93 (0.79, 1.10)	0.40
Average certification period length	1.12 (0.87, 1.45)	0.37
Vehicle exclusion from asset test	0.86 (0.73, 1.01)	0.06

a. Model parameters listed in Supplemental Methods. Study population is all low income (family income <185% of the federal poverty limit) households included in CPS. Person-level survey weights used. Clustered standard errors used to account for clustering of individuals within states. Food insecurity data obtained from the annual Current Population Survey Food Security Supplement.
b. The SNAP policy index is scaled from 0.1 to 10, with a higher value indicating greater adoption of policies associated with SNAP participation. Model includes baseline SNAP index (in 2009) and absolute change in SNAP index.
c. Single model with each policy included as covariates. The following policies were not included as there was no variation across the study period – proportion of SNAP benefits accounted for by EBT (electronic benefit transfer) and eligibility for legal noncitizen children (age < 18). Model includes baseline value for policies.

© 2025 Potluri S et al. *JAMA Health Forum*.
