# Supplementary material for: Supplemental Nutrition Assistance Program Policies and Food Insecurity
Source: JAMA Health Forum. 2025 Dec 12;6(12):e255597. doi: 10.1001/jamahealthforum.2025.5597 (PMC12701512; doi:10.1001/jamahealthforum.2025.5597)
Supplement: Supplement 2. — Data Sharing Statement [file jamahealthforum-e255597-s002.pdf]

Data Sharing Statement

Potluri. Supplemental Nutrition Assistance Program Policies and Food Insecurity. *JAMA Health Forum*. Published December 12, 2025. doi:10.1001/jamahealthforum.2025.5597

Data
Data available: No

Additional Information
Explanation for why data not available: All data used are publicly available
